# Programmed Cell Death Protein 1/Programmed Cell Death Ligand-1 Axis activates Intracellular ERK Signaling in Tumor Cells to Mediate Poor Prognosis in T-cell Lymphoma

**DOI:** 10.7150/jca.55971

**Published:** 2021-08-25

**Authors:** Yang Li, Yue Fei, Lu Liu, Zheng Song, Xiangrui Meng, Lihua Qiu, Lanfang Li, Zhengzi Qian, Shiyong Zhou, Xiubao Ren, Chengfeng Bi, Bin Meng, Huilai Zhang, Xianhuo Wang, Kai Fu

**Affiliations:** 1 Department of Lymphoma, Tianjin Medical University Cancer Institute and Hospital, National Clinical Research Center of Cancer, Key Laboratory of Cancer Prevention and Therapy, the Sino-US Center for Lymphoma and Leukemia Research, Tianjin, China.; 2 Department of Immunology/Biotherapy and Tianjin Medical University Cancer Institute and Hospital, National Clinical Research Center of Cancer, Key Laboratory of Cancer Prevention and Therapy, the Sino-US Center for Lymphoma and Leukemia Research, Tianjin, China.; 3 Department of Pathology and Microbiology, University of Nebraska Medical Center, Omaha, NE, USA.; 4 Pathology, Tianjin Medical University Cancer Institute and Hospital, National Clinical Research Center of Cancer, Key Laboratory of Cancer Prevention and Therapy, the Sino-US Center for Lymphoma and Leukemia Research, Tianjin, China.

**Keywords:** PD-L1, p-ERK, prognosis, T-cell lymphoma

## Abstract

**Purpose:** To investigate the prognostic significance of programmed cell death ligand-1 (PD-L1) and phosphorylated ERK (p-ERK) and their interactions in T-cell lymphoma (TCL).

**Methods:** The mRNA levels of PD-L1 and ERK in TCL samples were analyzed. Formalin-fixed paraffin-embedded tissues from 69 TCL patients were collected to detect the expression of PD-L1 and p-ERK by multiplexed immunofluorescence staining. The total PD-L1 and p-ERK was measured by western blotting, and membrane PD-L1 was determined using flow cytometry.

**Results:** PD-L1 and ERK mRNA levels were significantly upregulated in TCL. The expression rates of PD-L1 and p-ERK were 52.2% and 27.5%, respectively. PD-L1 expression correlated with stage (R=0.304, *P*=0.011) and IPI score (R=0.313, *P*=0.009), and p-ERK expression correlated with stage (R=0.330, *P*=0.006) and IPI score (R=0.376, *P*=0.002). PD-L1 expression positively correlated with p-ERK expression (R=0.355,* P*=0.003). Patients with co-expression of PD-L1 and p-ERK had the worst overall survival (*P*=0.007). In three TCL cell lines with PD-L1 expression, we demonstrated that the expression of p-ERK was upregulated after stimulation with PD-1, suggesting that ERK signaling was activated.

**Conclusions:** The PD-1/PD-L1 axis activates intracellular ERK signaling in tumor cells and that PD-L1, p-ERK or their combination are potential biomarkers for predicting the prognosis in TCL patients.

## Introduction

T-cell lymphoma (TCL) is a rare lymphoid malignancy. The incidence of TCLs is lower than that of B-cell lymphoma, by approximately 10-15%. TCLs are a broad group of heterogeneous diseases, and are mainly categorized into nodal TCLs, anaplastic large-cell lymphomas (ALCL), cytotoxic TCLs and leukemias, cutaneous T-cell lymphomas (CTCL), and EBV^+^ T-cell and NK-cell lymphomas 1. TCLs, except for anaplastic lymphoma kinase (ALK)-positive ALCL, have a poor prognosis because of their aggressive biological characteristics and resistance to therapy 2. According to the National Comprehensive Cancer Network (NCCN) guidelines for T-cell lymphoma (2018 version), clinical trials and multiagent chemotherapy are recommended as the treatments for TCL patients, except for those with ALK-positive ALCL and NT/T-cell lymphoma 3. Most patients are treated with CHOP/CHOP-like chemotherapy regimens. Five-year overall survival (OS) rates vary somewhat by subtype, with an average OS of 35% 4-6. The anthracycline-based chemotherapy regimens are still disappointing, and new therapy regimens are urgently needed. PD-1/PD-L1 inhibitors have gradually achieved gratifying results for the treatment of lymphomas 7, 8. The possibility of individualized immunotherapy holds promise, but advancements in research are still needed.

PD-L1 is an inhibitory T-lymphocyte receptor ligand that is expressed on hematopoietic and non-hematopoietic cells, such as T cells, B cells and various types of tumor cells. PD-L1 has specificity for binding to PD-1, which provides negative signals that control and inhibit T-cell responses, thus facilitating immune escape of tumor cells 9. The expression of PD-L1 in tumor cells has been linked to poor prognosis in a wide variety of cancers 10, 11. Many previous studies have focused more on the exhaustion of T cells, but there is a lack of research on whether PD-1/PD-L1 is directly involved in the regulation of tumor cell-intrinsic signals. PD-L1 is a type I transmembrane protein encoded by CD274 or B7-H1, with 290 amino acids, including extracellular immunoglobulin variable region (IgV) like domain, hydrophobic transmembrane domain and intracellular domain, suggesting its potential role in tumor immune evasion 12. Structural analysis shows that the intracellular domain consists of C-terminal and N-terminal amino acid residues and contains two independent phosphorylation sites, indicating that it may be involved in the activation of intracellular signal transduction, which has also been reported in previous studies 10. Thus, a better understanding of various immune-oncological pathways with therapeutic targets is necessary to design future trials that optimize the use of combination strategies for various lymphomas. It was reported that tumor cell-intrinsic PD-L1 promotes tumor-initiating cell generation and functions in melanoma and ovarian cancer 13, 14. Our previous study also demonstrated that the stimulation of PD-1 could activate the intracellular AKT/mTOR pathway by the PD-1/PD-L1 axis and that coexpression of PD-L1 and p-AKT is associated with poor prognosis in diffuse large B-cell lymphoma (DLBCL) 15. However, whether PD-1/PD-L1 can activate other signaling pathways remains unclear.

The p44/42 MAPK (ERK1/2) signaling pathway is a part of the mitogen-activated protein kinase (MAPK) family, which is a widely conserved family of serine/threonine protein kinases involved in many cellular programs, such as cell proliferation, differentiation, motility, and death 16. ERK1/2 is considered to be an important target in the diagnosis and treatment of cancer, and the first potent highly selective ERK inhibitors have now been developed and are entering clinical trials 17, 18. However, studies on ERK1/2 in TCL are rare.

The aims of this study were to assess the prognostic value of PD-L1 and phosphorylated ERK (p-ERK) expression in TCL and to investigate whether PD-1/PD-L1 binding could directly activate the intracellular ERK1/2 pathway in tumor cells.

## Materials and methods

### Microarray data

GEO (http://www.ncbi.nlm.nih.gov/geo) is a public functional genomics data repository of high throughout gene expression data, chips and microarrays 19. The gene expression dataset (GSE6338) 20 were downloaded from GEO (Affymetrix GPL570 platform, Affymetrix Human Genome U133 Plus 2.0 Array). The probes were converted into the corresponding gene symbol according to the annotation information in the platform. The GSE6338 dataset contained 20 normal T cells samples and 40 TCL samples.

### Patient samples

Our retrospective study included patients with de novo TCL newly diagnosed according to the criteria of World Health Organization (WHO) classification and further confirmed by two independent hematopathologists between December 2006 and April 2016 at the Tianjin Medical University Cancer Institute and Hospital (TMUCIH). Inclusion criteria included: age ≥ 18 years; complete laboratory examination; ≥ four cycles treatment with CHOP-like regiments, including CHOP, CHOPE, COP, DA-EPOCH, ENDOSTAR+CHOPT; adequate formalin-fixed paraffin-embedded (FFPE) tissues. Exclusion criteria included: patients with known or suspected CNS involvement; no prior systemic therapy for lymphoma; patients with secondary tumor. Sixty-nine TCL patients were finally enrolled in this study. FFPE tissues from these patients at the diagnosis were collected to detect the expression of PD-L1 and p-ERK. This study was approved by the Clinical Research Ethics Board of TMUCIH and conducted in accordance with the 1964 Declaration of Helsinki. Informed consent was obtained from all individual participants.

### Multiplexed immunofluorescence staining

Multiplexed immunofluorescence staining was performed to visualize the expression of PD-L1 and p-ERK using Opal immunostaining (Perkin Elmer, USA), similar as described in our previous study 21. The antibodies against PD-L1 and p-ERK were purchased from Cell Signaling Technology (CST#13684 and CST#4370, USA). Antibodies and fluorescent dyes (listed in order) were anti-PD-L1/Opal 570, anti-p-ERK/Opal 620. Specifically, we cut the paraffin tissue into 4 µm-5 µm sections. For each slide, we baked the slide in the oven at 70 °C for 1 hour, dewaxed it with xylene (3 × 10 min), and rehydrated it through a graded series of ethanol solutions (100% 1 × 10 min; 95% 1 × 10 min; and rinse in 70%). After rehydration, we briefly rinsed the slides in distilled water. We placed pH 6.0 citrate buffer in the microwave for 4 min at 100% power, and then placed the slides in the microwave for an additional 15 min at 20% power, and allowed them to cool down at room temperature before proceeding (1 h-1.5 h). We covered the tissue sections with blocking buffer and incubated them for 10 min at room temperature. We then incubated the slides with anti-PD-L1 antibodies overnight at 4 °C. We added a secondary antibody polymer with horseradish peroxidase (HRP) dropwise to the sections and incubated for 10 min at room temperature. We used opal-570 amplification reagent to incubate the slides at room temperature for 10 minutes with tyramide signal amplification (TSA) technology and pH 6.0 citrate buffer strips for the primary-secondary-HRP complex, allowing the introduction of the next anti-p-ERK antibodies. The corresponding amplification reagent was opal-620. After two sequential rounds of staining, the slides were counterstained with DAPI (Life Technologies), mounted with VectaShield HardSet medium (#1882542, Invitrogen, USA), and then stored in a light-proof box at 4 °C prior to imaging.

### Scoring for PD-L1 and p-ERK

A professional computer-assisted platform (Perkin Elmer) was used to assess the H-score of each FFPE tissue, which demonstrated marker staining automatically. Tumor and nonmalignant lymphoid cell staining for PD-L1 was considered positive if the H-score ≥30 of the cell population showed moderate or strong membrane staining. A patient was considered to be tumor tissue positive for p-ERK if the H-score was ≥10. The threshold used here is comparable to the median H-score of PD-L1 or p-ERK. Evaluation of immunofluorescence was performed independently by two observers without knowledge of patient outcome.

### Cell lines and flow cytometry

TCL cell lines, including Hut78, HH and Jurkat, were cultured in RPMI 1640 medium (Gibco, Thermo Fisher Scientific, USA) containing 10% fetal bovine serum (Gibco) and 1% penicillin/streptomycin (Gibco). The membrane PD-L1 (mPD-L1)expression on these cell lines was determined using flow cytometry. Briefly, cells were collected (1×10^6^ cells/100 µl), washed with PBS (pH 7.2), and then incubated with fluorochrome-conjugated PE anti-human PD-L1 (clone 29E.2A3, BioLegend, USA) for 30 min at 4 °C in a dark room. Then, mPD-L1 expression was measured by flow cytometry (LSRFortessa, BD Bioscience, USA) and repeated in triplicate. Data were analyzed with FlowJo software (version 7.6).

### Plasmids construction and establishment of Jurkat with knock-down of PD-L1

PD-L1 shRNA and control plasmids were constructed as following: Briefly, fragment containing coding PD-L1 was amplified from human genomic DNA using the following primers: 5'-CcggACCATCAAGTCCTGAGTGGTACTCGAGTACCACTCAGGACTTGATGGTTTTTTg-3' (forward) and 5'-aattcaaaaaACCATCAAGTCCTGAGTGGTACTCGAG TACCACTCAGGACTTGATGGT-3' (reverse). The PCR product was cloned into the GV644 vector (Genechem Co., Ltd. Shanghai, China) to generate the PD-L1 shRNA recombinant plasmid.

Plasmid transfection was performed with LV-gene (55263-1) Transfection reagent (Genechem Co., Ltd. Shanghai, China) according to the manufacturer's protocol. Briefly, lentivirus was packaged into HEK293T cells. Virus pseudovirus particles were collected to infect the Jurkat target cells to generate PD-L1 shRNA Jurkat cells. All transduced cells were selected with puromycin.

### Western blotting

Total PD-L1 expression in TCL cell lines was determined using western blotting similar to our previous study 15. After stimulating TCL cell lines with PD-1/Fc polypeptide (#10377-H03H, Sino Biological, Inc., USA), the levels of ERK and p-ERK were also measured using western blotting. The stimulated process was as follows: Briefly, TCL cell lines positive for mPD-L1 were stimulated by 0.24 µg/mL PD-1/Fc polypeptide for 0 h, 0.5 h, 1 h and 2 h at 37 °C, and then total protein was collected for detecting the expression of β-actin (#41554, GeneTex, USA), ERK (#4695, Cell Signaling Technology, USA) and p-ERK (#4370, Cell Signaling Technology, USA).

### Statistical analysis

The associations among PD-L1, p-ERK expression and clinicopathological characteristics were estimated with two-tailed Pearson analyses. Kaplan-Meier analyses were used to compare survival curves. Statistical analyses were performed using SPSS 22.0. A two-sided *P* value less than 0.05 was considered statistically significant.

## Results

### Overexpression of PD-L1 and ERK mRNA in TCL tissues

**Figure 1:**
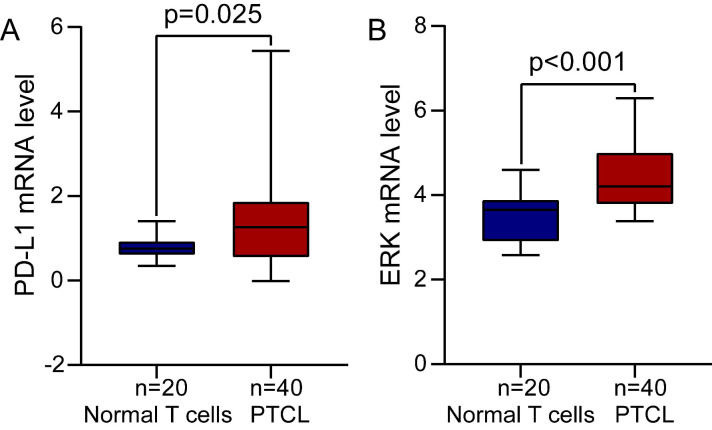
** The expression of PD-L1 and ERK mRNA in TCL tissues from GEO database. (A)** PD-L1 mRNA level was significantly increased in PTCL. **(B)** ERK mRNA level was significantly upregulated in PTCL.

We first analyzed the differential expression of PD-L1 mRNA in PTCL (one of TCL subtypes) samples and normal T cells samples from the GEO database and found that PD-L1 mRNA level was significantly increased in PTCL compared to normal T cells (Figure 1A, *P*=0.025). The differential expression of ERK mRNA in PTCL samples and normal T cells samples was also analyzed in the same way, and we found that ERK mRNA level was also significantly upregulated in PTCL as well (Figure 1B, *P*<0.001).

### Relationship between PD-L1 and p-ERK expression with clinical characteristics

The clinical characteristics of the 69 studied patients are summarized in Table 1, including 44 men and 25 women (median age 54 years, range 5-79 years old). According to the 2016 WHO classification, sixteen (23%) patients were diagnosed with ALCL, ten (14%) patients were diagnosed with angioimmunoblastic lymphoma (AITL), fourteen (20%) patients were diagnosed with peripheral T-cell lymphoma (PTCL) without classification, sixteen (23%) patients were diagnosed with peripheral T-cell lymphoma-not otherwise (PTCL-NOS) and thirteen (19%) patients were diagnosed with other rare and unclassifiable TCLs, including T-cell leukemia/lymphoma, enteropathy-type, subcutaneous panniculitis-like TCL. Kaplan-Meier survival curves showed that those patients with ALCL and other subtypes had a significantly superior OS than patients with AITL, PTCL-NOS and PTCL without classification subtypes (*P*=0.024; Figure S1). Twenty-five patients (36%) were classified as Ann Arbor clinical stages I-II disease, and 44 patients (64%) were classified as Ann Arbor clinical stages III-IV disease. Thirty-nine patients (57%) were evaluated as IPI 1-2 scores, and 30 patients (43%) were evaluated as IPI 3-4 scores. In total, 36 patients (52.2%) were PD-L1-positive cases, which correlated with stage and IPI score (R=0.304, *P*=0.011; R=0.313, *P*=0.009). The positive rate of PD-L1 in stage III-IV patients was higher than that in stage I-II patients (63.6% *vs* 32.0%) and the positive rate of PD-L1 in patients with IPI 3-4 was higher than that in patients with IPI 1-2 (70.0% *vs* 38.5%). In total, 19 patients (27.5%) were positive for p-ERK, which was associated with stage and IPI score (R=0.330, *P*=0.006; R=0.367, *P*=0.002). The positive rate of p-ERK in stage III-IV patients was higher than that in stage I-II patients (38.6% *vs* 8.0%). Similarly, the positive rate of p-ERK in patients with IPI 3-4 was higher than that in patients with IPI 1-2 (46.7% *vs* 12.8%).

### Correlation between PD-L1 and p-ERK expression

**Figure 2:**
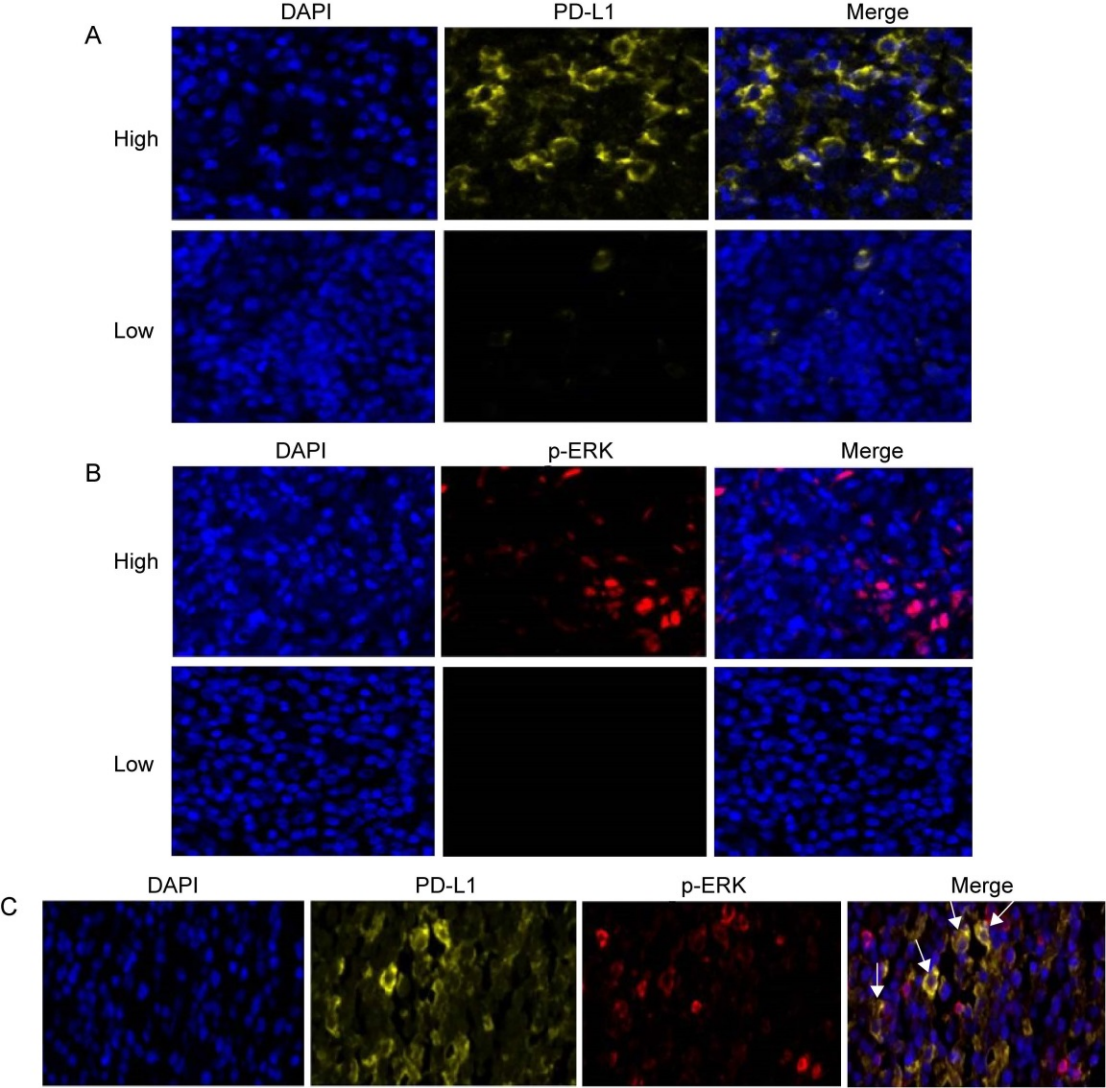
** Expression of PD-L1, p-ERK and their coexpression in TCL tissues. (A)** Representative patterns of high and low expression levels of PD-L1. **(B)** Representative patterns of high and low expression levels of p-ERK. **(C)** Representative coexpression patterns of PD-L1 and p-ERK (White arrows point to some coexpression cells of PD-L1 and p-ERK). DAPI (blue), PD-L1 (yellow) and p-ERK (red).

**Figure 3:**
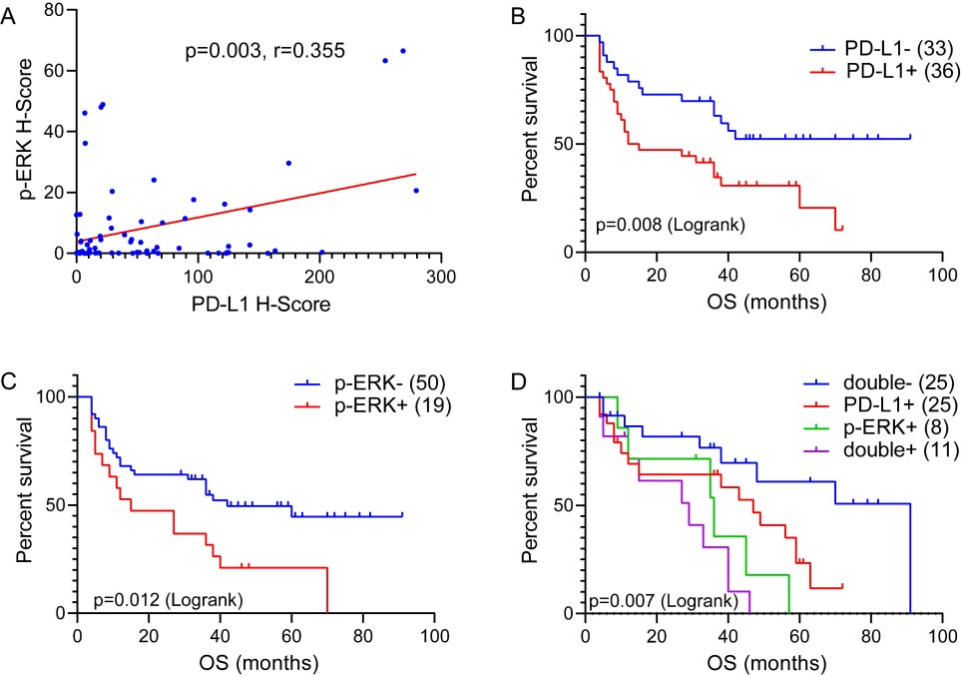
** The correlation between PD-L1 expression and p-ERK expression, and Overall survival (OS) of 69 TCL patients treated with CHOP-like regimen. (A)** The correlation between PD-L1 expression and p-ERK expression in 69 TCL tissues. **(B)** OS of TCL patients according to PD-L1 expression. **(C)** OS of TCL patients according to p-ERK expression. **(D)** OS of TCL patients according to the double-expression of PD-L1 and p-ERK.

Three-color multispectral images and separated individual spectral images within the same FFPE tumor section displayed that the staining pattern for PD-L1 protein was well defined in the plasma membrane, whereas staining for the p-ERK protein showed both nuclear and cytoplasmic staining patterns (Figure 2). An assessment of the association between PD-L1 expression and p-ERK expression in tumor tissues revealed that PD-L1 expression was positively correlated with p-ERK expression in TCL (Pearson's R=0.355,* P*= 0.003; Figure 3A).

### Double-expression of PD-L1 and p-ERK characterizes poorer outcomes in TCL

To further determine the effect of the PD-L1/p-ERK immunosuppressive axis in TCL, we studied whether the combination of PD-L1 expression and p-ERK expression in tumor tissues identified subgroups of patients with distinct clinical outcomes. All 69 patients were available for the OS analysis. The median OS for the full cohort was 35 months, ranging from 4 months to 91 months. Kaplan-Meier survival curves showed that PD-L1 and p-ERK expression levels corresponded with a significantly poorer OS than patients with negative PD-L1 and p-ERK expression in this cohort of patients (*P*=0.008, *P*=0.012; Figure 3B and C, respectively). Moreover, TCL patients with coexpression of PD-L1 and p-ERK had the worst OS compared to patients with single-positive or double-negative expression of PD-L1 and p-ERK (*P*=0.007, Figure 3D).

### Total PD-L1 and mPD-L1 expression in TCL cell lines

**Figure 4:**
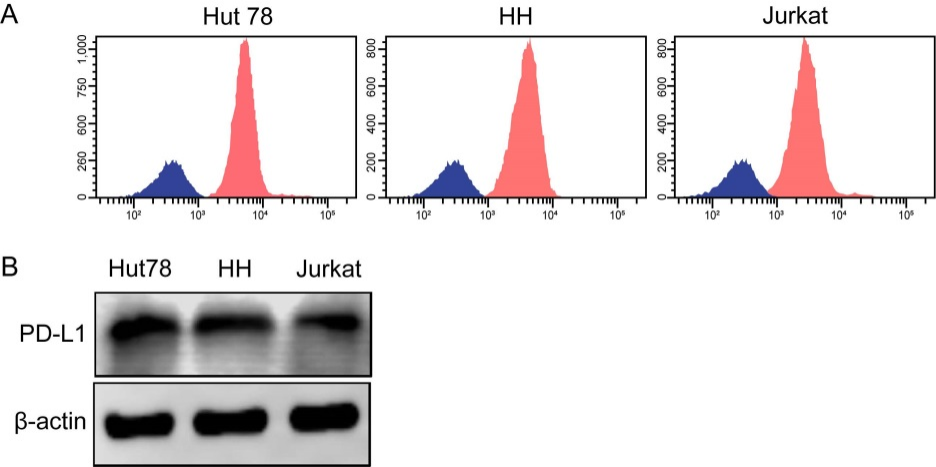
** PD-L1 and mPD-L1 are expressed widely in TCL cell lines. (A)** Total PD-L1 protein expression in TCL cell lines detected by western blotting. **(B)** Expression of mPD-L1 in TCL cell lines detected by flow cytometry.

Three human TCL cell lines, including Hut78, HH and Jurkat, were selected to detect total PD-L1 expression and mPD-L1 expression. All of the TCL cell lines exhibited high PD-L1 expression levels according to western blotting and flow cytometry (Figure 4A and B).

### PD-1/PD-L1 binding directly upregulated the intracellular p-ERK levels in TCL cells

**Figure 5:**
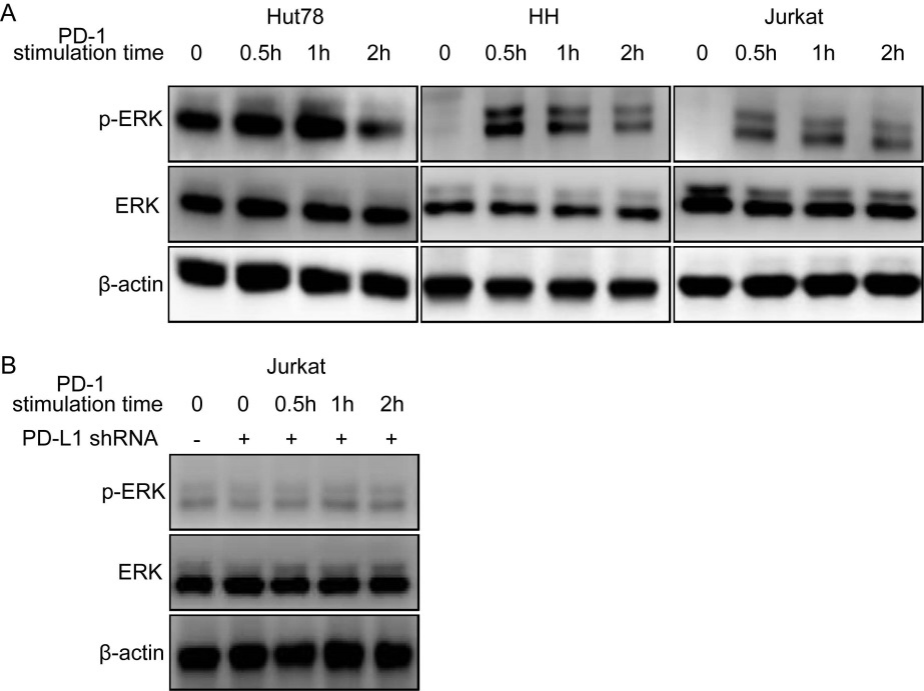
** PD-1/PD-L1 binding directly activates intracellular ERK1/2 oncogene signaling in TCL cell lines. (A)** ERK1/2 oncogene signaling is activated in Hut78, HH and Jurkat cell lines. **(B)** ERK1/2 oncogene signaling activation is blockaded after knock-down of PD-L1 in Jurkat cell lines.

To identify whether the binding of PD-1 and PD-L1 could activate the intracellular signaling in tumor cells, we used an activated PD-1 polypeptide to stimulate TCL cell lines for 30 min to 2 h and tested downstream phosphorylated protein levels by western blotting. The results revealed that the p-ERK level was significantly upregulated in the three TCL cell lines at 0.5-1h, indicating that ERK signaling was activated after treating cells with PD-1 (Figure 5A). The similar results were also observed in NKT cell lines (Figure S2). However, the effect was terminated when the PD-L1 on the TCL cell line was knocked-down (Figure 5B). In conclusion, these data demonstrated that tumor-intrinsic active phosphorylated protein could be activated directly through PD-1/PD-L1 in TCL cell lines.

## Discussion

Patients with ALK-positive ALCL seem to have a good response to a CHOP-like regimen, and the majority of natural killer/TCL (NKTL) patients localized to the nose and nasal sinuses are cured after the treatment of radiotherapy-containing regimens. However, first-line treatment regimens, including CHOP-like, offer poor efficacy and prognosis for other TCL patients, with a remission rate of only 50-65% 6, 22. Moreover, in patients with remission, the relapse is very common, and autologous stem cell transplantation is commonly used after the first remission but is limited by its own toxicity 23. In short, anthracycline-containing regimens have disappointing results for most TCL patients, and a new approach is needed.

Recent studies have shown more interest in the role of the PD-1/PD-L1 pathway in regulating the host antitumor response. As the major ligand of PD-1, PD-L1 is widely expressed and correlated with poor prognosis in many human cancers, which is in agreement with our results in this study. In our study, we found that nearly half of the TCL patients overexpressed PD-L1, and PD-L1-positive TCL patients had a poorer OS than the patients who had negative PD-L1 expression. PD-L1 expression was associated with poor survival in TCL patients. Thus, PD-1 or PD-L1 blockage might be a novel therapeutic approach for TCL patients. The blockade of PD-1/PD-L1 interaction using monoclonal antibodies has been approved by the US Food and Drug Administration (FDA) as an effective therapy for several solid tumors and further approved in Hodgkin lymphoma as the first hematological indication, and the significance of PD-1/PD-L1 blockade in TCL therapeutic strategy is still unclear.

While several important oncogenic signaling pathways that make TCL a heterogeneous disease and impact the OS of TCL patients have been verified, the MEK1/2-ERK1/2 pathway has not yet been reported in TCL. Activated ERK participates in multiple downstream pathways to promote the malignant phenotype of cancer cells by mediating various prosurvival signals, and is hence considered to be an important target in the diagnosis and treatment of cancer 24. In our study, we found that one-third of the patients had p-ERK overexpression and that the patients with positive expression of p-ERK had a more disappointing OS. The expression of phosphorylated-ERK1/2 (p-ERK1/2) was correlated with the survival of TCL patients.

In addition, we found that PD-L1 expression was positively correlated with p-ERK expression in TCLs, and patients with coexpression of PD-L1 and p-ERK had the worst OS compared to patients who were single-positive or double-negative for PD-L1 and p-ERK. Meanwhile, PD-L1 protein consists of immunoglobulin V-like and C like domains, a hydrophobic transmembrane domain, and a cytoplasmic tail domain, and has been confirmed that it has a potential to directly activate the intracellular oncogenic signaling pathways 15. ERK can be phosphorylated by the upstream activated molecules. Based on these results, we hypothesized that the expression of PD-L1 may interact with p-ERK. Therefore, we further selected three TCL cell lines with high PD-L1 and mPD-L1 expression, and then stimulated these cell lines using an activated PD-1 polypeptide for thirty minutes to two hours, and lastly detected the levels of ERK and p-ERK. Interestingly, we found that the level of total ERK protein was unchanged and p-ERK protein was significantly upregulated in a time-dependent manner. And p-ERK expression peaked at 0.5-1 h and then decreased gradually, suggesting that PD-1/PD-L1 binding could directly activate intracellular ERK oncogenic signaling in TCL tumor cells. These findings have been validated in other tumor cell lines. In conclusion, the PD-1/PD-L1 axis may positively promote ERK oncogenic signaling.

Our results indicated that the combination of PD-1/PD-L1 antibodies and ERK inhibitors might be a promising and novel therapeutic approach for TCL in the future. In addition, PD-L1 and p-ERK expression levels were all related to the stage and IPI score, which may result in poor OS in TCL patients. In our study, the number of patients included was relatively small, so a large cohort is required for further validation. However, this study showed a consistent trend that TCL patients with coexpression of p-ERK and PD-L1 had an even worse prognosis than patients with single-positive or double-negative expression of PD-L1 and p-ERK, which was treated with either CHOP or CHOP-like therapeutic strategy. These results suggest that the resistance of PD-L1 or p-ERK therapy may be an effective means to our antitumor therapy and that the combined usage of PD-L1 and p-ERK inhibitors may offer hope for cancer treatment.

Above all, TCL patients overexpressing PD-L1 and p-ERK showed significantly worse OS than the other TCL patients, and the coexpression of these proteins indicated a worse OS than that of patients with single-negative or double-negative expression levels. PD-1/PD-L1 binding might activate the intracellular ERK oncogenic signaling pathway in tumor cells to promote TCL aggressiveness. Thus, a more effective treatment approach should be developed for the TCL patients included in our study, and the combination of targeting ERK 1/2 and PD-1/PD-L1 pathway blockade is a promising therapeutic strategy.

## Supplementary Material

Supplementary figures.

## Figures and Tables

**Table 1:** Relationship between PD-L1 and p-ERK expression with clinical characteristics

Clinical characteristics	n	PD-L1 expression		Pearson	*P*	p-ERK expression		Pearson	*P*
		High (%)	Low (%)	R		High (%)	Low (%)	R	
Total	69	36 (52.2)	33 (47.8)			19 (27.5)	50 (72.5)		
**Gender**				-0.058	0.632			0.127	0.291
Male	44	22 (50.0)	22 (50.0)			14 (31.8)	30 (68.2)		
Female	25	14 (56.0)	11 (44.0)			5 (20.0)	20 (80.0)		
**Age**				0.528	0.076			0.142	0.237
<60	37	18 (48.6)	19 (51.4)			8 (21.6)	29 (78.4)		
≥60	32	18 (56.3)	14 (43.7)			11 (34.4)	21 (65.6)		
**B symptom**				-0.013	0.916			-0.071	0.558
No	36	19 (52.8)	17 (47.2)			11 (30.6)	25 (69.4)		
Yes	33	17 (51.5)	16 (48.5)			8 (24.2)	25 (75.8)		
**Stage**				0.304	0.011*			0.330*	0.006*
I-II	25	8 (32.0)	17 (68.0)			2 (8.0)	23 (92.0)		
III-IV	44	28 (63.6)	16 (36.4)			17 (38.6)	27 (61.4)		
**IPI score**				0.313	0.009*			0.376*	0.002*
1-2	39	15 (38.5)	24 (61.5)			5 (12.8)	34 (87.2)		
3-4	30	21 (70.0)	9 (30.0)			14 (46.7)	16 (53.3)		
**LDH**				0.104	0.390			0.059	0.622
<247	36	17 (47.2)	19 (52.8)			9 (25.0)	27 (75.0)		
≥247	33	19 (57.6)	14 (42.4)			10 (30.3)	23 (69.7)		
**β-microglobulin**				0.123	0.306			-0.075	0.532
Normal	25	11 (44.0)	14 (56.0)			8 (32.0)	17 (68.0)		
Elevated	44	25 (56.8)	19 (43.2)			11 (25.0)	33 (75.0)		
**Subtype**				2.960	0.564			9.257	0.055
AITL	10	5 (50.0)	5 (50.0)			5 (50.0)	5 (50.0)		
ALCL	16	7 (43.8)	9 (56.2)			0 (0)	16 (100)		
PTCL	14	9 (64.3)	5 (35.7)			5 (35.7)	9 (64.3)		
PTCL-NOS	16	10 (62.5)	6 (37.5)			5 (31.3)	11 (68.7)		
others	13	5 (38.5)	8 (61.5)			4 (30.8)	9 (69.2)		

*P<0.05, *R The correlation is significant at the *P* level. LDH, lactic-dehydrogenase; AITL, angioimmunoblastic lymphoma; ALCL, anaplastic large cell lymphoma; PTCL, peripheral T cell lymphoma; PTCL-NOS, peripheral T cell lymphoma-not otherwise; others, T-cell leukemia/lymphoma, enteropathy-type, subcutaneous panniculitis like and other rare T cell lymphoma.

## Abbreviations

PD-L1: programmed cell death ligand-1
p-ERK: phosphorylated ERK
TCL: T-cell lymphoma
PD-1: programmed cell death protein 1
ALCL: anaplastic large-cell lymphomas
CTCL: cutaneous T-cell lymphomas
ALK: anaplastic lymphoma kinase
NCCN: National Comprehensive Cancer Network
OS: overall survival
IgV: immunoglobulin variable
DLBCL: diffuse large B-cell lymphoma
MAPK: mitogen-activated protein kinase
TMUCIH: Tianjin Medical University Cancer Institute and Hospital
FFPE: Formalin-fixed paraffin-embedded
mPD-L1: membrane PD-L1
AITL: angioimmunoblastic lymphoma
PTCL: peripheral T-cell lymphoma
PTCL-NOS: peripheral T-cell lymphoma-not otherwise
NKTL: natural killer/TCL
FDA: Food and Drug Administration
