# Supplementary material for: Programmed Cell Death Protein 1/Programmed Cell Death Ligand-1 Axis activates Intracellular ERK Signaling in Tumor Cells to Mediate Poor Prognosis in T-cell Lymphoma
Source: J Cancer. 2021 Aug 25;12(20):6126–34. doi: 10.7150/jca.55971 (PMC8425197; doi:10.7150/jca.55971)
Supplement: Supplementary file 1 — Supplementary figures. [file jcav12p6126s1.pdf]

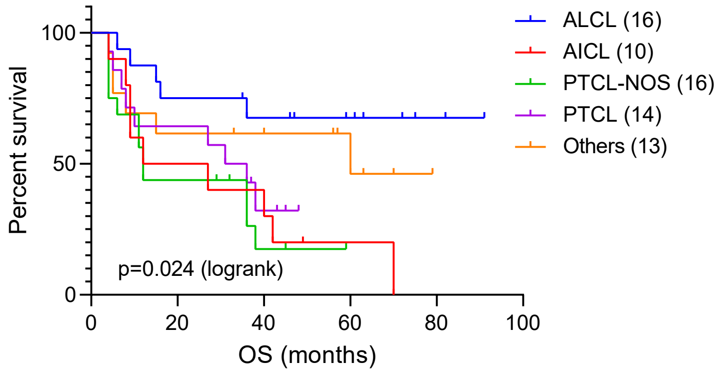

Figure S1. Overall survival (OS) of different subtypes of 69 TCL patients treated with CHOP-like regimens.

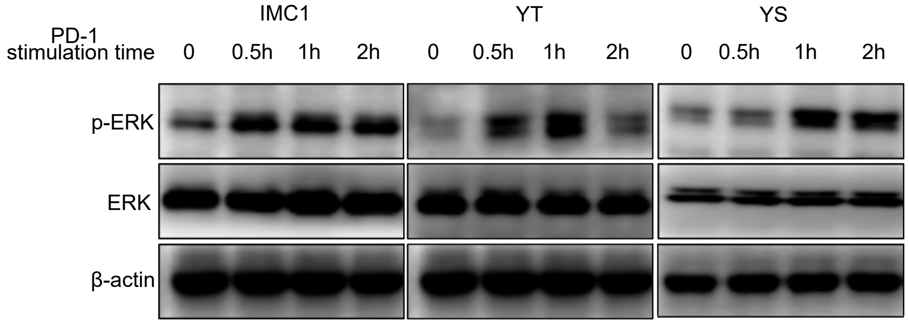

Figure S2. ERK1/2 oncogene signaling is activated in IMC1, YT and YS cell lines.
